# Impact of different CAD software programs on marginal and internal fit of provisional crowns: An in vitro study

**DOI:** 10.1016/j.heliyon.2024.e24205

**Published:** 2024-01-08

**Authors:** Ayşe Rençber Kızılkaya, Aybuke Kara

**Affiliations:** Department of Prosthodontics, Faculty of Dentistry, Firat University, Elazig, Turkey

**Keywords:** Dentbird, Exocad, Geomagic, Inlab, Internal clearance, Marginal fit, Software

## Abstract

**Statement of the problem:**

Different CAD software programs used for designing crowns show variations in marginal and internal fit. Marginal and internal discrepancies may cause poorly fitting crowns.

**Purpose:**

The aim of this study was to compare the marginal and internal fit of single crown temporary restorations designed using three different CAD software programs.

**Materials and methods:**

Dentbird, Exocad and Inlab 20 were used to design temporary single crowns using the same cement gap. Three experimental groups (n = 10/group) were formed based on the CAD software used. Geomagic Control X three-dimensional analysis software was used to compare the marginal and internal fit among the groups. Measurements were obtained at nine different thickness points. IBM SPSS Statistics, version 22 was used for all statistical analyses.

**Results:**

Among the CAD software programs tested, Dentbird produced the best internal fit on the buccal surface and the best marginal fit on both buccal and mesial surfaces. Exocad achieved the best values on the distal surface for both internal and marginal fit, while Inlab showed the best values on the mesial surface for internal fit and on the palatal surface for marginal fit.

**Conclusions:**

The Dentbird CAD software program provided the most accurate fit values that closely matched the design. The marginal and internal fit oftemporary crowns may vary depending on the CAD software used.

## Teaser

**CLINICAL IMPLICATIONS**: The marginal and internal fit of temporary crowns may show variations depending on the CAD software used for their design and this should be considered by clinicians. More successful clinical results can be achieved using the CAD software program that produces ideal marginal and internal fit.

## 1 Introduction

Recent technological advances led to the development of computer-aided manufacturing (CAM) and three-dimensional (3D) digital production systems for use in the field of dentistry. Design software programs have been introduced for this purpose. Technical errors that impact traditional workflows diminish the quality of prosthetics, and such errors can be minimized through a fully digital the workflow involving computer-aided design (CAD), obviating the need for numerous steps in dental laboratories [1,2].

Digital manufacturing technologies encompass equally important data acquisition, data processing, and production steps. Any error occurring during the process will result in fabrication of an ill-fitting permanent restoration. Hence, scanning, CAD, and manufacturing steps are the key determinants of marginal and internal fit of any restorations fabricated with CAM and 3D printing, which need to be evaluated [[3], [4], [5], [6], [7]].

CAD software programs can be classified as dental and open-source programs. Dental software programs offer straightforward scenarios for dental practitioners, yet their versatility in creating virtual designs is poor compared to open-source programs. Moreover, open-source software programs enable production of virtual designs with lower costs [8,9].

Digital dentistry employs a variety of software solutions to restore missing structures using different methodologies. Exocad (EXO; exocad GmbH) is one of the most widely used software for such purposes. Exocad offers different components that are either provided by default in the design or can be modified within a software environment [10].

The software defaults for crown design in the Exocad software may lead to formation of spaces between the inner surface of the crown and the outer surface of the prepared tooth. This can subsequently result in variable crown thickness in a standardized anatomy, affecting the compressive strength [11]. Cerec Inlab (IN; Dentsply Sirona) is another commonly used CAD software program. A tooth prepared with standard step-wise reduction is digitized using an optical scanner. Margins and contours are defined, followed by the manufacturing process [12].

More recently, Dentbird (Imagoworks Inc.), a dental CAD software program, was introduced. Although not yet widely used, Dentbird was utilized by Capobianco et al. to design molar restorations for calculating stress concentration under various conditions, based on crown restorative material and the presence of fatigue [13].

The Dentbird CAD software from Imagoworks offers a significant advantage for clinicians due to its easy accessibility and web-based nature. When using Dentbird, dental practitioner uploads the patient's 3D intra-oral scan to the software. The program's artificial intelligence (AI) automatically classifies the data, and crown design requires only a few additional clicks. Then, the AI instantly identifies the teeth that need restoration, determines the number of teeth for each case, scans margin lines, and designs the crowns accordingly. The software designs the crown that best fits the oral structure of individual patient, especially in terms of occlusion with the neighboring teeth, leaving little for dentists to make final adjustments [14].

When the long-term health of the tooth and surrounding tissues is considered, temporary restorations are equally important as permanent restorations. Inappropriate temporary restorations can compromise the health of both the prepared teeth and periodontal tissues, adversely affecting the success of the final restoration. Therefore, utmost care should be exercised when manufacturing provisional restorations, using appropriate materials and techniques [15].

In general, the fit of restorations depends on the production method used [16]. Three-dimensional printing has significantly reduced fit-related issues. Although some studies showed that the 3D printing method improves the fit of fabricated temporary crowns in various aspects, further clinical studies are warranted to corroborate this finding [17].

Appropriate internal clearance (IC) between the abutment tooth and the fixed prosthesis is required to achieve correct prosthesis fit. If the IC is too small, the prosthesis may not seat properly on the abutment tooth. Particularly in all-ceramic restorations, an excessive IC may significantly reduce retention of the prosthesis, eventually influencing flexural strength [18]. A systematic review of the studies examining the fit of CAD/CAM-milled prostheses showed that the IC is a key parameter for achieving proper fit for the prostheses designed in CAD programs [19].

Faulty marginal fit (MF) and IC have an adverse impact on the clinical durability of the fabricated all-ceramic restorations [20,21]. However, studies on the effect of different CAD software programs used to design dental restorations on MF and IC are scarce [22].

Prosthesis fit is evaluated using a number of methods including microphotography and light microscopy [20], silicone replicas [23,24], cross-sectional imaging [25], 3D analysis software with a triple-scan protocol [[26], [27], [28], [29], [30]], and micro-computed tomography (mCT) [31].

The purpose of this study was to compare the MF and IC fit of single crown temporary restorations designed using three different CAD software programs. The null hypothesis of this study was that there would be no difference in marginal and internal fit of single crown restorations designed using different CAD software programs.

## 2 Materials and methods

**Fig. 1:**
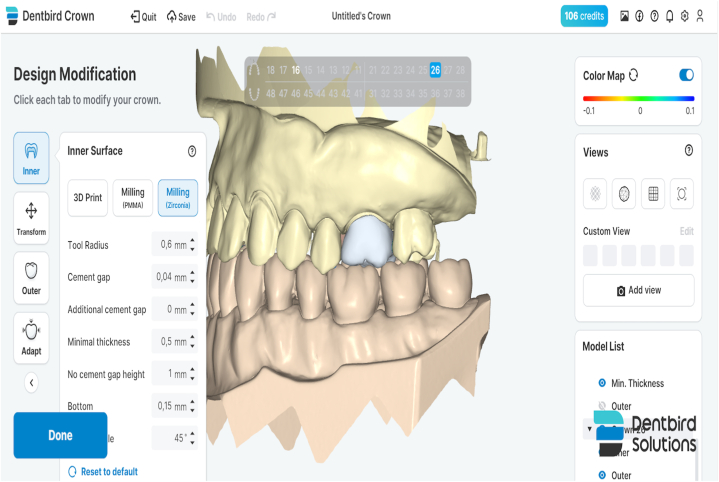
Crown design with Dentbird software.

**Fig. 2:**
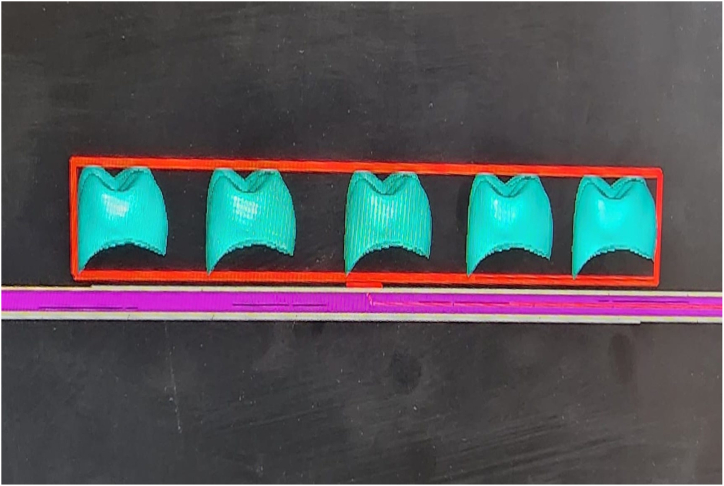
Crowns designed using the CAD software program.

**Fig. 3:**
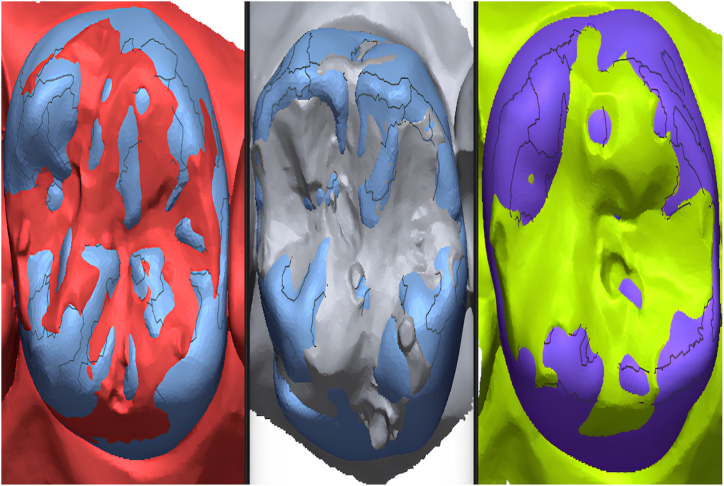
Superimposition of scanned models and main models using Geomagic Control X (3D Systems) 3D analysis software program (A: Dentbird, B:Exocad, C: Inlab).

**Fig. 4:**
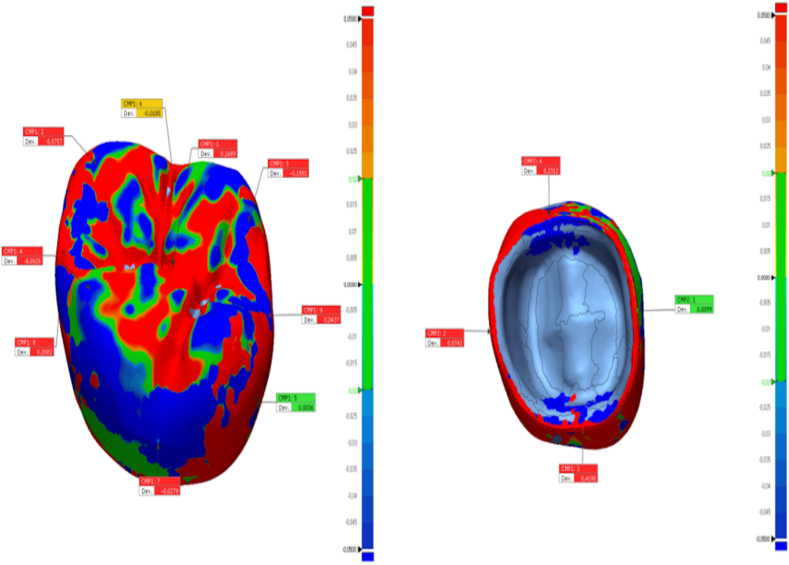
The points of measurement on the internal and marginal areas of the crowns designed using the Dentbird software.

**Fig. 5:**
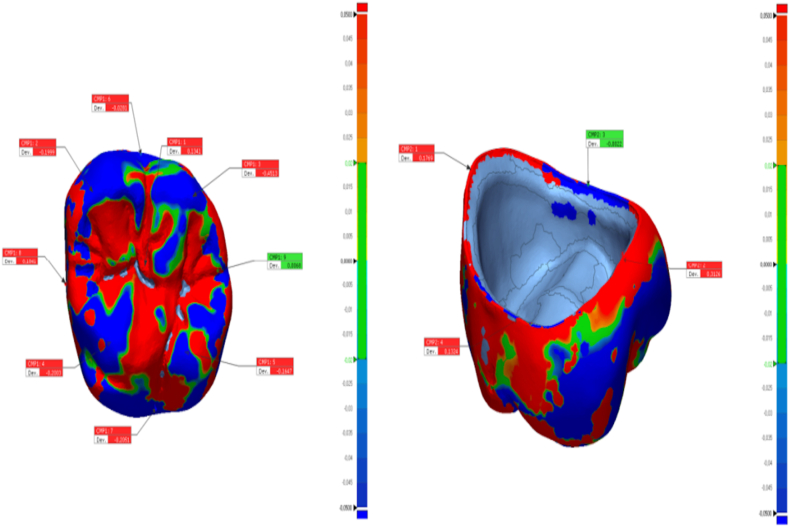
The points of measurement on the internal and marginal areas of the crowns designed using the Exocad software.

**Fig. 6:**
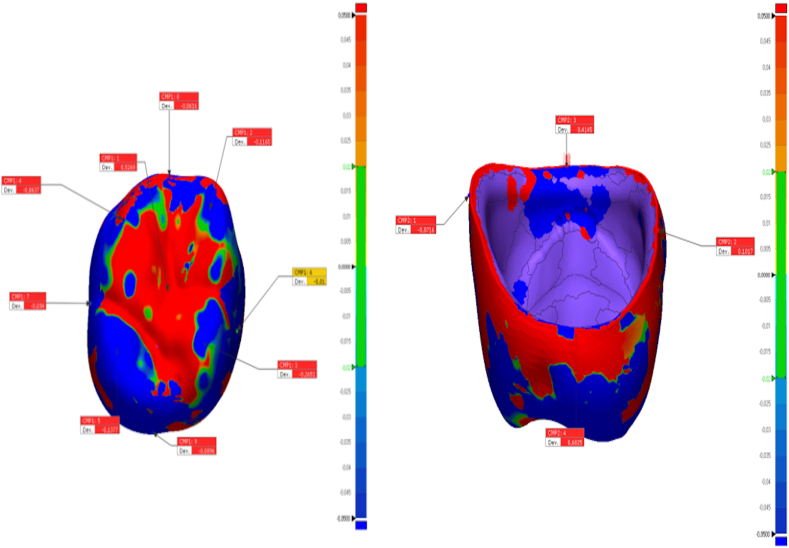
The points of measurement on the internal and marginal areas of the crowns designed using the Inlab 20 software.

In this study, the tooth #26 (FDI classification) of a ready-made mandibular model was prepared for the production of a temporary crown with a 1-mm chamfer finish line at the Department of Prosthodontics of Firat University Faculty of Dentistry. The tooth was scanned using a laboratory scanner (inEos X5; Dentsply Sirona). The resulting STL file was transferred to three different CAD software programs (Exocad, Dentbird, Inlab 20) for designing temporary crowns. You log in to the Dentbird system (Fig. 1) via the web and click "new" for the new design. Lower and upper jaw files are uploaded and the system adjusts automatic closing. For design, first material selection and parameters are set. Prepared tooth and step lines are automatically detected and corrections are made for minimal changes. The digit can be moved up and down with a single button (redetect margin line). By clicking "Generate crown", a crown is automatically created, modifications are made for minimal changes, and the design is completed and exported. While designing the temporary crowns, a standard cement gap parameter (0.40 mm) was set. The crowns designed with the three different CAD software programs were exported as STL files (Fig. 2). Production was carried out using a 3D printer (DentaFab) for each STL file, resulting in a total of 30 temporary crowns (n = 10 each). Liquid Temp Resin (3D printing Resin for Temporary teeth) was used for temporary crown production. Temporary crowns fabricated with DentaFab were cemented onto the prepared model using Cavex temporary cement. After cementation, the models were rescanned and exported in STL format. To evaluate MF and IC fit, the models scanned were superimposed on the main models designed in CAD design programs using Geomagic Control X (3D Systems) 3D analysis software program (Fig. 3). MF and IC were evaluated at 9 different points (occlusal surface, buccal wall, palatal wall, mesial wall, distal wall, buccal margin, palatal margin, mesial margin, distal margin) (Fig. 4, Fig. 5, Fig. 6) and the tolerance amount was set at ±0.02.

IBM SPSS Statistics, version 22 (IBM Corp.) was used for statistical analysis of the study data. The normality of data distribution was checked using Shapiro-Wilk test, revealing a non-normal distribution. Kruskal-Wallis test was used for comparisons of the study parameters among the groups, and the group causing the difference was identified using Dunn's test. The significance level was set at p < 0.05.

## 3 Results

### 3.1 Assessment of internal fit

A comparison of the internal fit values among the groups is presented in Table 1.

**Table 1:** Evaluation of internal fit.

Internal (mm)	Dentbird (n = 10)		Exocad (n = 10)		Inlab (n = 10)		
	Min/Max	Mean ± SD (median)	Min/Max	Mean ± SD (median)	Min/Max	Mean ± SD (median)	p
Occlusal	0.08/0.28	0.21 ± 0.09 (0.26)	0.11/0.46	0.21 ± 0.14 (0.16)	0.31/0.54	0.47 ± 0.1 (0.52)	0.018*
Buccal	−0.02/0.04	0.01 ± 0.02 (0)	−0.08/-0.02	−0.03 ± 0.02 (−0.03)	−0.05/0	−0.01 ± 0.02 (−0.01)	0.027*
Palatal	−0.09/-0.01	−0.06 ± 0.04 (−0.08)	−0.21/-0.04	−0.09 ± 0.07 (−0.06)	−0.07/-0.03	−0.05 ± 0.01 (−0.05)	0.613
Mesial	−0.05/0.21	0.06 ± 0.1 (0.02)	0.06/0.12	0.09 ± 0.03 (0.09)	−0.06/0.1	0.02 ± 0.07 (0.01)	0.289
Distal	0/0.18	0.07 ± 0.07 (0.05)	−0.03/0.03	0.01 ± 0.02 (0.01)	−0.15/-0.09	−0.12 ± 0.03 (−0.13)	0.004*

Kruskal-Wallis test *p < 0.05.

A statistically significant difference was observed among the groups in terms of the IC fit values for the occlusal surface (p < 0.05). Post hoc analyses were conducted to determine the group causing the significant difference, which showed that the IC fit values for the occlusal surface were significantly greater in the Inlab group versus the Exocad group (p < 0.05). There was no significant difference among the other groups (p > 0.05).

The IC fit values on the buccal surface differed significantly among the groups (p < 0.05). Post hoc analyses revealed that the IC fit values for the buccal surface were significantly greater in the Dentbird group compared to the Exocad group (p < 0.05). No significant difference was found among the other groups (p > 0.05).

For the IC fit values on the distal surface, a significant difference was found among the groups (p < 0.05). Post hoc analyses showed significantly greater IC fit values for the distal surface in the Dentbird group versus the Inlab group (p < 0.05). The difference in buccal IC values among the other groups was nonsignificant (p > 0.05).

The IC fit values for the palatal and mesial surfaces were not significantly different among the groups (both p > 0.05).

### 3.2 Assessment of marginal fit

Table 2 displays a comparison of marginal fit values among the groups.

**Table 2:** Evaluation of marginal fit.

Marginal (mm)	Dentbird (n = 10)		Exocad (n = 10)		Inlab (n = 10)		
	Min/Max	Mean ± SD (median)	Min/Max	Mean ± SD (median)	Min/Max	Mean ± SD (median)	p
Buccal	−0.16/0.05	−0.04 ± 0.08 (−0.02)	−0.05/0.25	0.12 ± 0.11 (0.15)	−0.07/0.1	0.03 ± 0.07 (0.04)	0.090
Palatal	0.04/0.13	0.08 ± 0.04 (0.07)	0.15/0.33	0.25 ± 0.08 (0.29)	−0.08/0.1	0.01 ± 0.09 (−0.02)	0.006*
Mesial	−0.03/0.42	0.16 ± 0.22 (0.02)	−0.09/0.38	0.17 ± 0.21 (0.23)	0.16/0.41	0.31 ± 0.1 (0.35)	0.512
Distal	0.23/0.6	0.38 ± 0.14 (0.34)	0.04/0.46	0.17 ± 0.17 (0.13)	0.4/0.68	0.55 ± 0.12 (0.55)	0.018*

Kruskal-Wallis test *p < 0.05.

There was a statistically significant difference among the groups in terms of the palatal MF values (p < 0.05). Post hoc analyses showed that the Exocad group had significantly greater palatal MF values than the Inlab group (p < 0.05). No significant difference was found among the other groups (p > 0.05).).

The MF values for the distal surface differed significantly among the groups (p < 0.05). Post hoc analyses revealed that the MF values for the distal surface were significantly greater in the Inlab group compared to the Exocad group (p < 0.05). There was no significant difference among the other groups (p > 0.05).

The groups did not exhibit a significant difference with respect to the MF values for the buccal and mesial surfaces (both p > 0.05).

## 4 Discussion

In our study comparing the MF and IC fit of single crown temporary restorations designed using three different CAD software programs, our null hypothesis was that there would be no difference in MF and IC fit of single crown restorations when designed with different CAD software programs. However, based on the study results, significant differences emerged among the groups, leading to the rejection of our hypothesis.

Studies have been conducted on the MF and IC of fixed prostheses fabricated using CAD/CAM methods. Many of these studies have chosen 3D scanners as an independent variable and MF and IC as dependent variables [[32], [33], [34]]. However, previous studies have not taken into consideration how different CAD software programs and CAM systems might influence the production of fixed prostheses. Moreover, studies investigating the accuracy of CAD/CAM-milled prostheses found significant differences between the designed and final prostheses [35].

In a study by Reich et al., the MF and IC of all-ceramic three-unit fixed dentures were compared with those of metal-ceramic fixed dentures using the replica technique. Three different CAD/CAM systems (Digident, Lava, and Cerec Inlab) were used to fabricate the ceramic frameworks. The median MF values were 75 μm for Digident, and 65 μm for both Lava and Inlab. The median IC values were 94 μm for Digident, 105 μm for Lava, 154 μm for Cerec Inlab, and 75 μm for metal-ceramic dentures. The replica thickness values for the occlusal area were 326 μm for Digident, 198 μm for Lava, 359 μm for Cerec Inlab, and 287 μm for metal-framework ceramic [36]. In the current study, we compared three different CAD software programs, and for the IC fit, Exocad (−0.16 mm) produced the optimal median value on the occlusal surface, Dentbird (0 mm) on the buccal surface, Inlab (−0.05 mm) on the palatal surface, Inlab (0.01 mm) on the mesial surface, and Exocad (0.01 mm) on the distal surface. For MF, Dentbird (−0.02 mm) achieved an ideal median value on the buccal surface, Inlab (−0.02 mm) on the palatal surface, Dentbird (0.02 mm) on the mesial surface, and Exocad (0.13 mm) on the distal surface.

Lee et al. designed a three-unit fixed partial denture using three different CAD software programs (Exocad, Dental SystemTM, and inLab 16) and employed Geomagic Control (Geomagic Inc., 3D Systems), a 3D analysis software, to calculate the IC error. The accuracy of the computer-aided design varied depending on the type of CAD software used. Dental System™ achieved the best accuracy in the margins, axial walls, and occlusal surfaces, followed by Exocad and inLab 16 [22]. Similarly, in our study, we compared the MF and IC fit of crowns fabricated using three different CAD software programs. The same 3D surface scanning measurement technique was used. Dentbird provided the best accuracy in both marginal and internal areas, while Exocad and Inlab exhibited almost identical accuracy.

A study by Shimizu et al. compared the MF and IC values among the crowns fabricated using CAD/CAM systems with different scanners. In that study, a 3D analysis software was used to evaluate the fit of digital crowns manufactured using intra-oral scanners and CAD programs compared to those produced using extra-oral scanners and CAD programs. The MF and IC fit of the digital crowns fabricated using intra-oral scanners and CAD programs were found to be inferior compared to those fabricated using extra-oral scanners and CAD programs [35]. In our study, Inlab showed the highest MF value on the distal surface and the highest IC fit value on the occlusal surface. On the other hand, Dentbird exhibited the lowest MF value on the mesial surface and the lowest IC fit value on the distal surface.

Shim et al. compared the MF and IC of prostheses using two different versions of a CAD program for a single CAD/CAM system. Initially, the researchers set the IC parameter to either 40 μm or 80 μm and observed that when the IC parameter was set to 80 μm, the actual prostheses fit better [4]. In our study, we compared the MF and IC parameters using different CAD software programs, assuming a fixed cement gap of 40 μm.

In a study by Sarhan et al., single crowns with a similar cement gap were fabricated using three different CAD software programs (Exocad, Dental System, and B4D) and a 3D analysis software was used for evaluation. The accuracy of the cement gap parameter in single crown design varied depending on the CAD software used. The highest accuracy was achieved with the Dental System software on all dental surfaces, followed by B4D on the tooth margin and axial wall, and Exocad on the occlusal surface [37]. In the present study, even though we used the same cement gap parameter (40 μm), optimal IC and MF values were observed with Dentbird software, followed by Exocad software, across all dental surfaces.

In Bayrak et al.’s study, three different CAD software programs (CEREC, KaVo, and Planmeca) were used to design ceramic fillings, with the cement gap set at 80 μm for occlusal and axial walls and 25 μm for the marginal edge. The IC fit of the designed ceramic fillings was examined using the micro-CT method, and then all designs were milled on the same milling machine. They found that the CAD software program used affected the IC fit of ceramic fillings, with significant differences found among some of the groups. Tukey's post hoc analysis showed that the KaVo design values were greater than those of CEREC. Linear measurements revealed that the CEREC design values were greater versus the other two designs. There was no statistically significant difference between the Planmeca and KaVo groups [38]. In our study, optimal IC fit values were observed with Dentbird and Exocad software, while the Dentbird software achieved ideal MF values as well.

In a study by Al Haideri et al., two different in-lab CAD software designs (Exocad, 3Shape) were used to design monolithic zirconia crowns, and the MF and IC fit were evaluated using a sectioning procedure. The study showed that the crown MF area was comparable in both software designs but significant differences were observed in the IC area [39]. Likewise, a 3D surface inspection technique was used in our study. MF values differed significantly between Exocad and Inlab on the palatal and distal surfaces, while IC values showed significant differences between Inlab and Exocad on the occlusal surface, Dentbird versus Exocad on the buccal surface, and Dentbird versus Inlab on the distal surface.

Capobianco et al. designed molar restorations using Dentbird to evaluate fracture and stress distribution under different conditions based on crown restorative material and the presence of fatigue. The study concluded that feldspathic and leucite-based ceramics are weaker than lithium disilicate but their fracture resistance remained stable after fatigue. Lithium disilicate exhibited the strongest physicomechanical properties, bearing more stress during the fracture event [13]. In the current study, provisional crowns were designed using the Dentbird CAD software program, and MF and IC fit values were evaluated using a 3D surface scanning technique. The best fit values were observed for the Dentbird software program. Since the Dentbird is not yet widely used, further studies involving comparisons with other software programs are needed to confirm our results.

The limitations of this study are acknowledged. The points of measurement have not been fully standardized. Studies comparing the MF and IC values among crowns designed using different CAD software programs are limited in number. The materials produced using the 3D technology are still being developed, and therefore, further studies are warranted to identify the software programs that produce the best fitting restorations.

## 5 Conclusions

The following conclusions have been drawn from this study.

1. The software programs of different CAD systems affect the MF and IC fit of crowns produced.
2. New software programs show promise for providing user-friendly experiences.

## Data availability statement

Data will be made available on request.

## CRediT authorship contribution statement

**Ayşe Rençber Kızılkaya:** Writing - original draft, Visualization, Conceptualization. **Aybuke Kara:** Writing - original draft.

## Declaration of competing interest

The authors declare that they have no known competing financial interests or personal relationships that could have appeared to influence the work reported in this paper.
